# Effects of CTLA4-Ig treatment on circulating fibrocytes and skin fibroblasts from the same systemic sclerosis patients: an in vitro assay

**DOI:** 10.1186/s13075-018-1652-6

**Published:** 2018-07-27

**Authors:** Maurizio Cutolo, Stefano Soldano, Paola Montagna, Amelia Chiara Trombetta, Paola Contini, Barbara Ruaro, Alberto Sulli, Stefano Scabini, Emanuela Stratta, Sabrina Paolino, Carmen Pizzorni, Vanessa Smith, Renata Brizzolara

**Affiliations:** 1 Research Laboratory and Academic Division of Clinical Rheumatology, Department of Internal Medicine, University of Genoa, IRCCS San Martino Polyclinic Hospital, Viale Benedetto XV, 616132 Genoa, Italy; 2 Division of Clinical Immunology, Department of Internal Medicine, University of Genoa, Genoa, Italy; 3 Oncologic Surgery, Department of Surgery, IRCCS San Martino Polyclinic, Genoa, Italy; 4 Department of Rheumatology, Ghent University Hospital, Ghent University, Ghent, Belgium

**Keywords:** Fibrocytes, Skin fibroblasts, CTLA4-Ig, Systemic sclerosis, Connective tissue disease

## Abstract

**Background:**

Systemic sclerosis (SSc) is characterized by vasculopathy and progressive fibrosis. CTLA4-Ig (abatacept) is able to interact with the cell surface costimulatory molecule CD86 and downregulate the target cell. The aim of this study was to evaluate the in-vitro effects of CTLA4-Ig treatment on circulating fibrocytes and skin fibroblasts isolated from the same SSc patient.

**Methods:**

Circulating fibrocytes and skin fibroblasts were obtained from eight SSc patients with “limited” cutaneous involvement and from four healthy subjects (HSs). Samples were analyzed by fluorescence-activated cell sorter analysis (FACS) at baseline (T0) and after 8 days of culture (T8) for CD45, collagen type I (COL I), CXCR4, CD14, CD86, and HLA-DRII expression. Circulating fibrocytes were treated for 3 h and skin fibroblasts for 24/48 h with CTLA4-Ig (10, 50, 100, 500 μg/ml). Quantitative real-time polymerase chain reaction (qRT-PCR) was performed for CD86, COL I, FN, TGFβ, αSMA, S100A4, CXCR2, CXCR4, CD11a, and Western blotting was performed for COL I and FN.

**Results:**

Using qRT-PCR, the T8-cultured SSc circulating fibrocytes which had not been treated with CTLA4-Ig showed higher gene expression for CD86, αSMA, S100A4, TGFβ, and COL I compared with HS circulating fibrocytes. Interestingly, αSMA/COL I gene expression was significantly lower only in the SSc circulating fibrocytes treated with CTLA4-Ig for 3 h (*p* < 0.01, *p* < 0.05). On the contrary, no effects were observed for either SSc or HS skin fibroblasts after CTLA4-Ig treatment. COL I and FN protein expression was unchanged in both SSc and HS skin fibroblasts by Western blot.

**Conclusions:**

Circulating fibrocytes seem to be more responsive to CTLA4-Ig treatment than skin fibroblasts from the same SSc patient, likely due to their higher expression of CD86. CTLA4-Ig treatment might downregulate the fibrotic process in SSc patients by downregulating the fibrocytes, circulating progenitor cells.

**Electronic supplementary material:**

The online version of this article (10.1186/s13075-018-1652-6) contains supplementary material, which is available to authorized users.

## Key messages

Circulating fibrocytes seem to be more responsive to CTLA4-Ig (abatacept) treatment than skin fibroblasts isolated from the same SSc patients affected by limited or diffuse cutaneous involvement.

The described effetct exerted by abatacept may represent a new approach for early intervention in SSc, therefore acting on progenitor cells (fibrocytes) before their final homing and differentiation into active myofibroblasts.

A new therapeutic option for abatacept in SSc treatment should be taken into consideration based on its possible antifibrotic effect.

## Background

Systemic sclerosis (SSc) is a systemic autoimmune connective tissue disease of complex etiology, characterized by microvasculopathy and progressive fibrosis [1, 2].

Activation of the immune response through autoantibody production, together with the recruitment and transition of endothelial cells and pericytes into active myofibroblasts, seems to play an important role in the progression of fibrosis in almost all organs. Therefore, although the pathogenesis of SSc remains unclear, myofibroblast activation is believed to be the final step following microvascular damage [3, 4]. Myofibroblasts are characterized by a higher expression of specific phenotype markers and profibrotic molecules, primarily α-smooth muscle actin (αSMA) and fibroblast-specific protein-1 (S100A4), as well as by the overproduction of extracellular matrix (ECM) proteins such as fibronectin (FN) and fibrillar collagens (type I and III) [5–7].

Various cell types, including endothelial cells, circulating mesenchymal cells, and even fibrocytes, may differentiate into myofibroblasts [8].

Fibrocytes are circulating progenitor cells derived from the bone marrow that express specific markers of both hematopoietic cells (CD34, CD43, CD45, LSP-1, and MHC class II) and stromal cells (collagen I and III), together with the chemokine receptors CCR2, CCR7, and CXCR4, which regulate their migration into inflammatory lesions [9–13]. Circulating fibrocytes are recruited through CXCR4/CXCL12 interaction into injured tissues where they differentiate into fibroblasts/myofibroblasts, thereby regulating the healing process (by producing cytokines, chemokines, and growth factors), secreting essential ECM proteins, and promoting angiogenesis [14–16].

Moreover, although fibrocytes are involved in physiological wound repair to local tissue injury, in chronic fibroproliferative disorders they may be the cause of excess deposition of ECM molecules [17].

In vitro, fibrocytes appear to differentiate from circulating CD14^+^ monocytes into spindle-shaped, fibroblast-like cells and seem to have an antigen-presenting capability, expressing class II major histocompatibility complex molecules (HLA-DP, -DQ, and -DR), the CD86 (B7.2) costimulatory molecule, and the CD11a, CD54 (ICAM-1: intracellular adhesion molecule-1), and CD58 adhesion molecules [9, 18–20]. When cultured in the presence of a specific antigen, human fibrocytes induce antigen-presenting cell (APC)-dependent T-cell proliferation which is significantly higher than that induced by monocytes and nearly as high as the proliferation of purified dendritic cells [20].

The costimulatory molecule CD86 is expressed on APCs, including macrophages, and the CTLA4-Ig fusion protein induces a significant downregulation of both proinflammatory cytokines (interleukin (IL)-6, tumor necrosis factor (TNF)α, and IL-1β) and transforming growth factor (TGF)β in cultured human macrophages [21]. Of note, these anti-inflammatory effects induced by the binding between CTLA4-Ig and CD86 on the macrophage surface are evident both in the presence and in the absence of T cells, indicating a direct action of CTLA4-Ig on APCs [21–23].

In addition, CTLA4-Ig interacts with the costimulatory molecule CD86 on human endothelial cells, masking its expression and modulating the expression of vascular endothelial growth factor receptor (VEGFR)-2 and ICAM-1, two important molecules involved in inflammatory and angiogenic processes that characterize several autoimmune diseases, including SSc [24].

Current treatment for SSc includes vasodilators, disease-modifying antirheumatic drugs (DMARDs), and immunosuppressive drugs, but with limited success. New approaches for the treatment of SSc and its fibrotizing processes are under investigation, including the use of CTLA4-Ig (abatacept) [25–27].

Since human fibrocytes seem to have an antigen-presenting capability, and would appear to be an important source of fibroblasts/myofibroblasts in the physiological and pathological tissue remodeling that characterizes SSc, the aim of this study was to isolate and culture human circulating fibrocytes and skin fibroblasts from the same SSc patients as well as from healthy subjects (HSs) to investigate the possible effects exerted in vitro by CTLA4-Ig treatment.

## Methods

### SSc patients and healthy subjects

Eight SSc patients (seven females and one male, mean age 65 ± 7 years) with “limited” cutaneous involvement (lSSc) and an “active” nailfold videocapillaroscopic (NVC) pattern of microvascular damage were recruited from the Division of Rheumatology at the University of Genova. Four age-matched HSs (three females, one male) were enrolled from the Department of Surgery of the IRCCS San Martino Hospital in Genoa during routine diagnostic procedures.

All enrolled SSc patients fulfilled the 2013 European League Against Rheumatism/American College of Rheumatology (EULAR/ACR) criteria for the diagnosis of SSc [28]. No evident clinical SSc complications were present at the time of skin sampling, and the patients were receiving treatment with vasodilators alone (mainly cyclic prostanoids). At the site of skin biopsy (forearm), the local average value of the modified Rodnan skin score (mRSS) was found to be equal to 1 [29].

All SSc patients and HSs provided informed consent and the study was approved by the local ethics committee (protocol number 273-REG-2015).

### Cell culture and treatments

Fibrocytes were isolated from the peripheral blood mononuclear cells (PBMCs) by centrifugation over Ficoll-Paque (Sigma-Aldrich) according to the manufacturer’s instructions. The cells were cultured at baseline (T0) on fibronectin-coated plates in Dulbecco’s modified Eagle’s medium (DMEM) with 20% fetal bovine serum (FBS), 1% penicillin-streptomycin, and 1% l-glutamine (Sigma-Aldrich) at 37 °C and 5% of CO_2._ After overnight culture, the nonadherent cells were removed by a single gentle aspiration, while adherent fibrocytes were cultured for a further 8 days (T8) [30].

Fibrocytes at T8 were cultured for 3 h with or without CTLA4-Ig at various concentrations (10, 50, 100, and 500 μg/ml) in accordance with previous in-vitro studies [21, 22, 24].

Skin fibroblasts were isolated from the full thickness biopsies that had been carried out on the involved skin at one-third of the distal forearm of SSc patients and of HSs, in accordance with the EUSTAR protocol and the Declaration of Helsinki [31].

After fibroblast expansion, skin fragments were removed to allow cell growth. Fibroblasts that were collected between the third and fifth culture passage were cultured for 24 and 48 h in the absence or in the presence of various concentrations of CTLA4-Ig (10, 50, 100, and 500 μg/ml).

### Fluorescence-activated cell sorter (FACS) analysis

After 8 days of culture (T8), adherent fibrocytes were lifted by incubation in ice-cold 0.05% EDTA in phosphate-buffered saline (PBS), and cell viability was determined by the trypan blue exclusion test.

Characterization and identification of fibrocytes was performed at T0 and T8 by FACS (Beckman Coulter Company) using anti-CD45 (anti-CD45-krome orange, Beckman Coulter Company), anti-COL I (anti-COL I-FITC, Milli-Mark, Millipore), anti-CXCR4 (anti-CXCR4-PE, Beckman Coulter Company), anti-CD14, anti-CD86, and anti-HLA-DRII monoclonal antibodies (anti-CD14-alexa Fluor 750, anti-CD86-PC7, and anti-HLA-DRII-PC5.5, Beckman Coulter Company) [30].

Relevant isotype controls for each monoclonal antibody were used in the initial setup and frequently between tests.

### Quantitative real-time polymerase chain reaction (qRT-PCR)

Fibrocytes were cultured for 8 days whereas skin fibroblasts were cultured up to 80% confluency prior to treatment with CTLA4-Ig, as described in the “Cell culture and treatments” section above.

Total RNA was extracted with NucleoSpin RNA/protein (Macherey-Nagel) and quantified by NanoDrop (Thermo Scientific), which also evaluates RNA integrity, in accordance with the manufacturer’s instructions. For each experimental condition, first-strand cDNA was synthesized from 1 μg total RNA using the QuantiTect Reverse Transcription Kit (Qiagen).

qRT-PCR was performed on an Eppendorf Realplex 4 Mastercycler using the Real MasterMix SYBR Green detection system (Eppendorf) in a total volume of 10 μl loaded in triplicate. Primers for CD86 (NM_175862.4), COL I (NM_000088), FN (NM_002026), TGFβ (NM_000660), αSMA (NM_001613), S100A4 (NM_002961), CXCR2 (NM_00116829), CXCR4 (NM_00100854), CD11a (NM_00111438), and β-actin (NM_001101, housekeeping gene) were supplied by Primerdesign.

Gene expression values were calculated using the comparative ΔΔCT method and they corresponded to the expression level (fold-increase) of the target gene compared with the calibrator sample (untreated cells) taken as the unit value by definition [32]. In each qRT-PCR assay, the melting curve was performed to confirm the specificity of the SYBR green assay.

### Western blotting

Skin fibroblasts were cultured to 80% confluency and treated as described in the “Cell culture and treatments” section above. At the end of treatment (24 and 48 h), cells were lysed with NucleoSpin RNA/protein (Macherey-Nagel). Protein quantification was performed by the Bradford method. For each experimental condition, 20 μg protein was separated by electrophoresis on Tris-Glycine gel and transferred onto Hybond-C-nitrocellulose membranes (Life Technologies Ltd.).

After 1 h in blocking solution (PBS 1× 0.1% triton-X, and 5% nonfat powdered milk) membranes were incubated overnight at 4 °C with the following primary antibodies: anti-human COL I (dilution 1:400, Vinci-Biochem) and FN (dilution 1:1000, Sigma-Aldrich). Membranes were also incubated with primary horseradish peroxidase (HRP)-conjugated antibody to human actin (dilution 1:10,000, Santa-Cruz Biotechnology) to confirm similar loading of protein samples onto the gels and the efficiency of the electrophoretic transfer.

Membranes were subsequently incubated with the following secondary antibodies: anti-rabbit IgG for COL I (dilution 1:2000, Cell Signaling Technology) and anti-mouse IgG for FN (dilution 1:1000, Cell Signaling Technology). Protein synthesis was detected using the enhanced chemiluminescence system (Luminata Crescendo, Millipore). Densitometric analysis was performed by UVITEC Analysis Software (UVITEC Cambridge).

For each experimental condition, the values of collagen type I (COL I) and FN synthesis were normalized to those of the corresponding actin. The resulting values of each treatment were compared with those of the untreated cells (CNT; taken as the unit value by definition) to obtain the level of protein synthesis.

### Statistical analysis

Statistical analysis was carried out by the nonparametric Mann-Whitney *U* test to compare unpaired treatment group data. Any *p* value below 0.05 was considered statistically significant. The final results of FACS, qRT-PCR, and Western blotting were the mean of the results obtained from the independent experiments performed on in-vitro cultures of fibrocytes and skin fibroblasts isolated from each SSc patient and HS. The results are reported as mean ± standard deviation (SD).

## Results

### FACS analysis

**Fig. 1:**
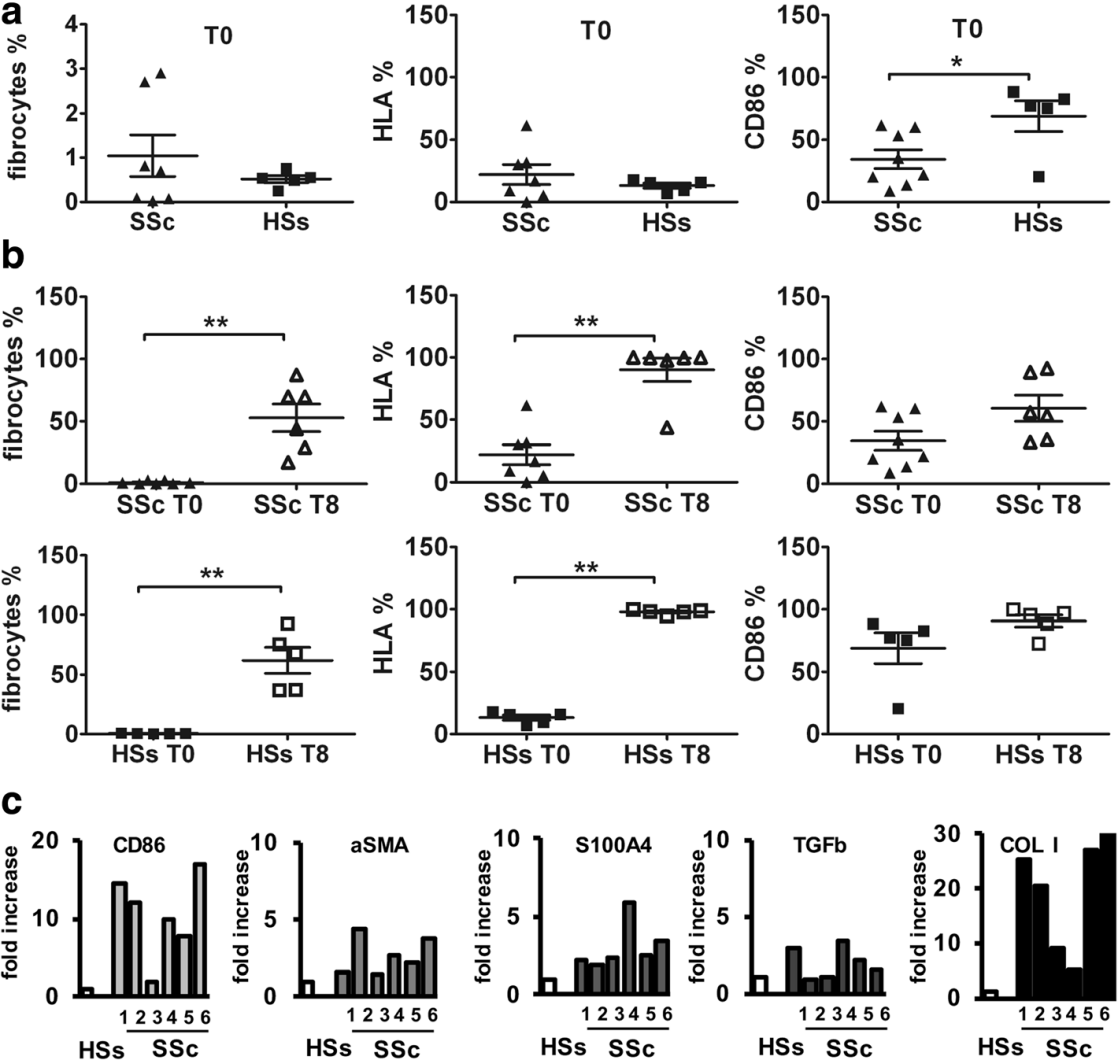
Characterization of systemic sclerosis (SSc) and healthy subject (HS) fibrocytes at basal time (T0) and at 8 days of culture (T8). **a** FACS analysis of SSc and HS fibrocytes, identified among the CD45^+^ cells, as CD45^+^, COL I^+^, CXCR4^+^, and relative HLA-DR and CD86 expression at T0; **b** FACS analysis of SSc and HS fibrocytes, identified among the CD45^+^ cells, as CD45^+^, COL I^+^, CXCR4^+^, and relative HLA-DR and CD86 expression at T0 and T8. **c** Quantitative RT-PCR analysis for CD86, αSMA, S100A4, TGFβ, and COL I gene expression of cultured SSc fibrocytes (T8), compared with HS fibrocytes (T8), taken as the calibrator

FACS analysis showed that at T0 the percentage of fibrocytes, identified as CD45^+^COL I^+^CXCR4^+^ cells, was 1.0 ± 1.2% in SSc patients and 0.5 ± 0.2% in HSs (50% less) (Fig. 1a). Moreover, in this fibrocyte population, the percentage of HLA-DR^+^ cells was very low (22.1 ± 21.1% and 13.1 ± 4.7%, respectively), whereas the percentage of CD86^+^ cells was higher in both SSc patients and HSs at T0 (34.4 ± 21.4% and 68.9 ± 27.6%) (Fig. 1a).

At T8, fibrocytes showed an adherent spindle-shaped morphology, and FACS analysis demonstrated that the percentage of CD45^+^COL I^+^CXCR4^+^ fibrocytes was significantly higher in both SSc patients and in HSs compared with T0 (up to 52.8 ± 27.1% vs. 1.0 ± 1.2% and up to 61.9 ± 24.4% vs. 0.5 ± 0.2%, respectively) (*p* < 0.01) (Fig. 1b).

At the same time, in this fibrocyte population, the HLA-DR^+^ cells were significantly increased in SSc patients and HSs compared with T0 (90.1 ± 22.7% vs. 22.1 ± 21.1% and 97.9 ± 1.9 vs 13.1 ± 4.7%, respectively) (*p* < 0.01) (Fig. 1b).

Similarly, the percentage of CD86^+^ fibrocytes was higher in SSc patients and HSs compared with T0 (60.4 ± 25.6% vs. 34.4 ± 21.4%, and 90.7 ± 10.9% vs. 68.9 ± 27.6%, respectively) with a greater increment in SSc fibrocytes (Fig. 1b).

### Quantitative real-time PCR

#### SSc fibrocytes

At T8, in the absence of CTLA4-Ig, SSc fibrocytes showed higher gene expression levels of CD86, αSMA, S100A4, TGFβ, and COL I compared with HS fibrocytes (Fig. 1c).

**Fig. 2:**
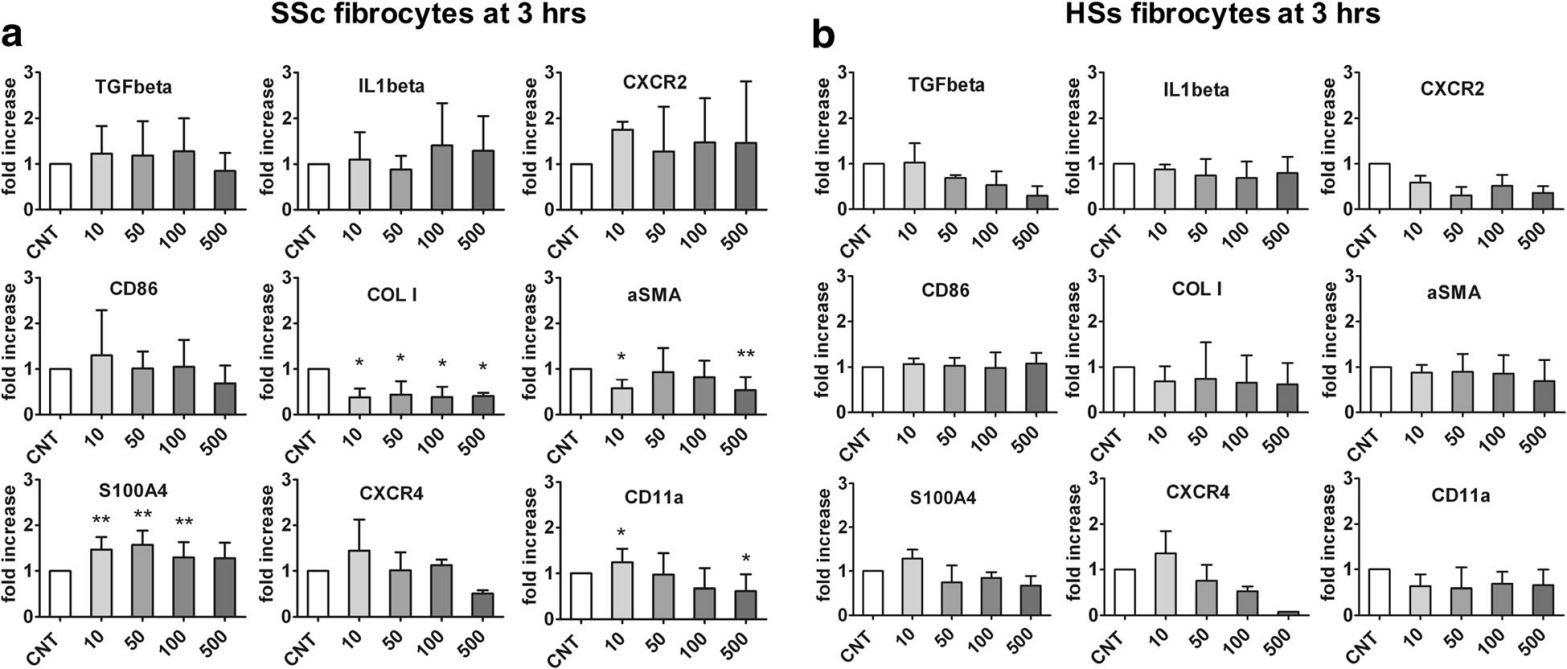
Quantitative RT-PCR analysis for TGFβ, IL-1β, CXCR2, CD86, COL I, αSMA, S100A4, CXCR4, and CD11a gene expression in cultures of systemic sclerosis (SSc) and healthy subject (HS) fibrocytes after 3 h of CTLA4-Ig treatment. Quantitative RT-PCR analysis for TGFβ, IL-1β, CXCR2, CD86, COL I, αSMA, S100A4, CXCR4, and CD11a gene expression in cultures of SSc fibrocytes (**a**) and HS fibrocytes (**b**) either untreated (CNT) or treated for 3 h with CTLA4-Ig at various doses (10, 50, 100, and 500 μg/ml). *p < 0.05, **p < 0.01

The SSc fibrocytes treated for 3 h with various concentrations of CTLA4-Ig (10, 50, 100, and 500 μg/ml) did not show any significant variations in the gene expression levels of TGFβ, IL-1β, and CXCR2 compared with CNT (Fig. 2a). In these cells, CD86 gene expression decreased (not significantly) after treatment with CTLA4-Ig 500 μg/ml (Fig. 2a).

Interestingly, the gene expression of COL I was significantly lower in SSc fibrocytes treated with CTLA4-Ig even at 10 μg/ml compared with CNT (*p* < 0.05) (Fig. 2a). Of note, αSMA gene expression also decreased after CTLA4-Ig treatment (significantly after CTLA4-Ig 10 μg/ml treatment, *p* < 0.05, and CTLA4-Ig 500 μg/ml treatment, *p* < 0.01), whereas S100A4 gene expression was significantly higher compared with CNT (*p* < 0.01) excluding at the concentration of 500 μg/ml (Fig. 2a).

Moreover, while treatment with CTLA4-Ig 500 μg/ml did not significantly reduce the gene expression of CXCR4, it did significantly reduce that of CD11a as compared with CNT (*p* < 0.05) (Fig. 2a).

#### HS fibrocytes

Unlike SSc fibrocytes, HS fibrocytes treated for 3 h with various concentrations of CTLA4-Ig (10, 50, 100, and 500 μg/ml) did not show any significant modulation in the gene expression levels of CD86 (Fig. 2b).

In addition, gene expression levels of TGFβ, CXCR2, COL I, CXCR4, and CD11a remained unchanged after CTLA4-Ig treatment compared with CNT, as did gene expression of αSMA and S100A4 (Fig. 2b).

#### SSc fibroblasts

Cultured SSc fibroblasts showed very low gene expression levels of CD86 compared with cultured macrophages obtained from the PBMCs of SSc patients, which were taken as positive controls for CD86 expression (Additional file 1).

**Fig. 3:**
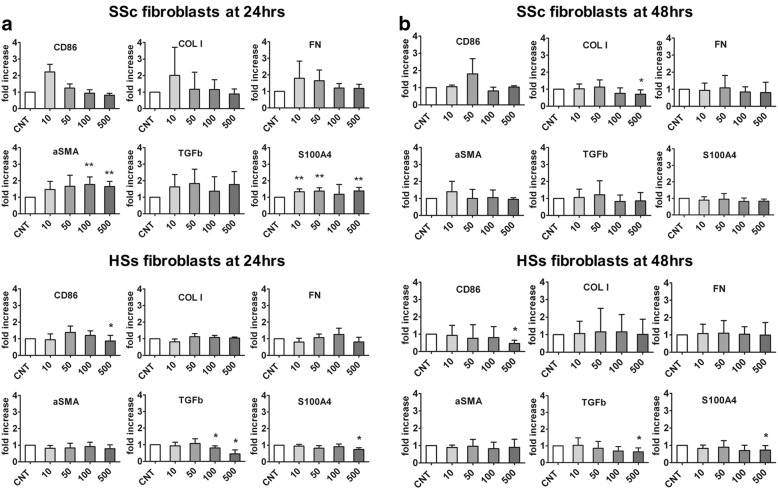
Quantitative RT-PCR analysis for CD86, COL I, FN, αSMA, TGFβ, and S100A4 gene expression in cultured systemic sclerosis (SSc) and healthy subject (HS) fibroblasts after 24 and 48 h of CTLA4-Ig treatments. Quantitative RT-PCR analysis for CD86, COL I, FN, αSMA, TGFβ, and S100A4 gene expression in cultures of SSc and HS fibroblasts either untreated (CNT) or treated for 24 h (**a**) and 48 h (**b**) with CTLA4-Ig at various doses (10, 50, 100, and 500 μg/ml). *p < 0.05, **p < 0.01

Nevertheless, cultured SSc fibroblasts treated for 24 h with CTLA4-Ig (10, 50, 100, and 500 μg/ml) did not show any significant differences in gene expression levels of CD86 compared with CNT (a nonsignificant increase after treatment with CTLA4-Ig 10 μg/ml was observed) (Fig. 3a). At the same time, gene expression of COL I and FN was higher only in the cultured SSc fibroblasts which had been treated with the lowest concentration of CTLA4-Ig (10 μg/ml) (Fig. 3a).

In addition, in these cultured CTLA4-Ig-treated SSc cells, the gene expression levels of αSMA, TGFβ. and S100A4 were higher compared with CNT (significantly for αSMA and S100A4; *p* < 0.01) (Fig. 3a).

On the other hand, the cultured SSc fibroblasts treated for 48 h with CTLA4-Ig (10, 50, 100, and 500 μg/ml) did not show any significant modulation in the gene expression levels of CD86, FN, αSMA, TGFβ, and S100A4 compared with CNT, whereas the gene expression of COL I was significantly downregulated by the highest dose of CTLA4-Ig (*p* < 0.05) (Fig. 3b).

#### HS fibroblasts

Cultured HS fibroblasts treated with CTLA4-Ig for 24 h showed a significant decrease in the gene expression of CD86, although this was limited to the highest dose (500 μg/ml) compared with CNT (*p* < 0.05) (Fig. 3a).

Of note, the gene expression of TGFβ and S100A4 was significantly reduced by the higher doses of CTLA4-Ig (*p* < 0.05 after 100 and 500 μg/ml for TGFβ; *p* < 0.05 after 500 μg/ml for S100A4) (Fig. 3a).

Similar to the results obtained after 24 h of CTLA4-Ig treatment, cultured HS fibroblasts showed a significant decrease in the gene expression levels of CD86 compared with CNT after 48 h of treatment with CTLA4-Ig, though this was limited to the highest dose (500 μg/ml) (*p* < 0.05) (Fig. 3b).

After 48 h of treatment with the highest dose of CTLA4-Ig (500 μg/ml), gene expression of TGFβ and S100A4 was significantly reduced compared with CNT (both *p* < 0.05) (Fig. 3b).

Gene expression of COL I, FN, and αSMA was unchanged after both 24 and 48 h of CTLA4-Ig treatment in cultured HS fibroblasts (Fig. 3a, b).

### Western blotting

**Fig. 4:**
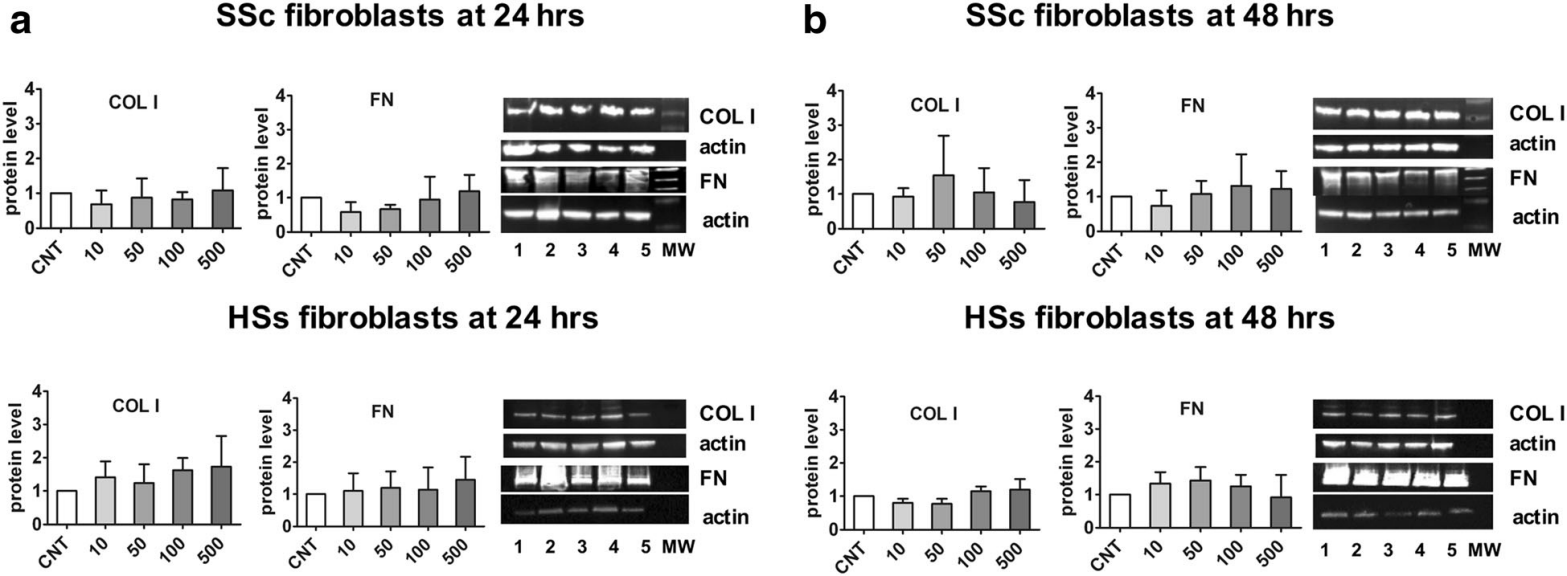
Western blot analysis of COL I and FN on cultured systemic sclerosis (SSc) and healthy subject (HS) fibroblasts after 24 and 48 h of CTLA4-Ig treatment. Western blotting analysis of COL I and FN in cultured SSc and HS fibroblasts. Western blotting of COL I and FN, and related densitometric analysis on cultured SSc and HS fibroblasts either untreated (CNT) or treated for 24 h (**a**) and 48 h (**b**) with various concentrations of CTLA4-Ig (line 1: CNT, calibrator; line 2: CTLA4-Ig 10 μg/ml: line 3: CTLA4-Ig 50 μg/ml; line 4: CTLA4-Ig 100 μg/ml; line 5: CTLA4-Ig 500 μg/ml; line 6: Molecular Weight)

The protein expression of COL I and FN in cultured SSc and HS fibroblasts was unchanged even after treatment with CTLA4-Ig at both 24 and 48 h compared with CNT (Fig. 4).

## Discussion

The present study reports for the first time that SSc circulating fibrocytes show an increased basal expression of αSMA and COL I compared with HS fibrocytes, suggesting their possible propensity for transition into activated myofibroblasts which are key cells involved in both tissue repair and fibrosis. Moreover, SSc circulating fibrocytes show a higher gene expression of CD86 and seem to be more responsive to treatment with the CTLA4-Ig fusion protein compared with the skin fibroblasts obtained from the same SSc patients.

This study also confirms that the percentage of circulating fibrocytes, characterized as CD45^+^COL I^+^CXCR4^+^ cells, was at least twice as high in SSc patients compared with HSs, although these cells are a minor component of the circulating pool of cells [11, 16, 33].

On the basis of these characteristics, we observed that once circulating fibrocytes are cultured in vitro they become adherent, develop a spindle-shaped morphology, and maintain the expression of key fibrocyte phenotype markers (CD45, COL I, and CXCR4), with a further increase in CD86 and HLA-DRII expression.

The interaction between the CTLA4-Ig fusion molecule (abatacept) and CD86 expressed on circulating fibrocytes is believed to alter and interfere with the function of these cells under pathological conditions, such as SSc, and in particular with their activation and differentiation into fibroblasts/myofibroblasts. The decrease in the gene expression of the main phenotypic markers of activated fibrocytes (COL I and CXCR4), together with the decreased gene expression of the myofibroblast phenotype marker αSMA induced by CTLA4-Ig treatment, suggests a possible downregulatory effect on the fibrocyte-myofibroblast transition process. It is interesting to note that these effects were evident in SSc circulating fibrocytes, but they were not observed in the HS circulating fibrocytes.

It is believed that, in response to injurious and inflammatory stimuli, human CD45^+^COL I^+^CXCR4^+^ fibrocytes traffic through the bloodstream and may be recruited into, and activated within, the injured and inflamed tissues where the chemokine receptors CXCR4 and CCR7 are reported to be pivotal [10, 14]. Of note, the ability of CTLA4-Ig to trigger a decrease in the expression of CXCR4 and CD11a adhesion/migration molecules on SSc circulating fibrocytes may suggest its possible action in interfering with trafficking and migration of these cells into inflammatory/altered sites [10, 14].

Fibrocytes can also function as APCs for the activation of CD8^+^ T cells by expressing major histocompatibility complex class I and II molecules and the costimulatory proteins CD80 and CD86 [20]. By binding to SSc circulating fibrocytes expressing CD86, CTLA4-Ig might interfere with their APC activity and could likely prevent the activation of T lymphocytes, as already demonstrated for other cellular targets (dendritic cells, B lymphocytes, macrophages, osteoclasts, endothelial cells) [21, 24, 34–37].

It is possible that, in SSc, circulating fibrocytes are already activated by the immune-inflammatory response associated with the disease, and that they are more responsive to other protein interactions, in particular to CTLA4-Ig, as compared with HS circulating fibrocytes.

A limitation of these in-vitro experiments relates to the small number of circulating fibrocytes that can be obtained from SSc patients and from HSs; therefore, larger samples are needed to confirm the obtained data. A further study evaluating SSc patients with diffuse skin involvement is already in progress. Very preliminary data obtained from in-vitro experiments on circulating fibrocytes isolated from only two patients, and treated with CTLA4-Ig, seem to show results similar to that described in our study.

Concerning fibroblasts, CD86 gene expression was found to be very low in cultured SSc fibroblasts, contrary to that found in circulating fibrocytes from the same patients, as already reported for murine fibroblasts [38]. As a possible consequence of the limited interaction with CD86, in the present short-term study (24 and 48 h) the CTLA4-Ig treatment did not induce a decrease in the ECM protein synthesis (COL I and FN) in cultured SSc fibroblasts or in HS fibroblasts.

A further study evaluating SSc patients with diffuse cutaneous involvement (dcSSc) is already in progress. Very preliminary data obtained from in-vitro experiments on circulating fibrocytes isolated from only two dcSSc patients, and treated with CTLA4-Ig, seem to show results similar to those described in lcSSc.

In addition, the histopathological literature in SSc attests that both the limited cutaneous as well as the diffuse cutaneous subset are characterized by the presence of myofibroblasts [39]. Hence, our study design should be equally applicable in limited cutaneous as well as diffuse cutaneous SSc.

Of note, the CTLA4-Ig treatment (at high doses) seems to induce a decrease in the TGFβ gene expression that can already be observed after 24 h of treatment; this, however, is limited to the cultured HS fibroblasts.

## Conclusions

In conclusion, circulating fibrocytes seem to be more responsive to CTLA4-Ig treatment than skin fibroblasts isolated from the same SSc patients characterized by limited cutaneous involvement. Thus, a new therapeutic option for abatacept in SSc treatment should be taken into consideration based on its possible antifibrotic effect.

Therefore, as described for the first time in the present study, the higher efficacy exerted at the gene expression level by CTLA4-Ig in cultured circulating fibrocytes versus cultured skin fibroblasts/myofibroblasts isolated from the same SSc patients may represent a new approach for early intervention, acting on progenitor cells before their final homing and differentiation into active myofibroblasts.

## Additional file

Additional file 1: **Figure S1.** CD86 gene expression levels in cultured SSc fibroblasts. Bar graph of quantitative RT-PCR analysis for CD86 gene expression in cultures of SSc fibroblasts, compared with SSc macrophages, taken as calibrator. (TIF 180 kb)

## Abbreviations

APC: Antigen-presenting cell
CNT: Untreated cells
COL I: Collagen type I
dcSSc: Diffuse cutaneous systemic sclerosis
DMARD: Disease-modifying antirheumatic drug
ECM: Extracellular matrix
EULAR/ACR: European League Against Rheumatism/American College of Rheumatology
FACS: Fluorescence-activated cell sorter
FN: Fibronectin
HS: Healthy subject
ICAM: Intracellular adhesion molecule
IL: Interleukin
lSSc: Limited systemic sclerosis
NVC: Nailfold videocapillaroscopy
PBMC: Peripheral blood mononuclear cell
PBS: Phosphate-buffered saline
qRT-PCR: Quantitative real-time polymerase chain reaction
S100A4: Fibroblast-specific protein-1
SMA: Smooth muscle actin
SSc: Systemic sclerosis
T*x*: Day *x*
TGF: Transforming growth factor
TNF: Tumor necrosis factor
VEGFR: Vascular endothelial growth factor receptor
