# Supplementary figures and images for: Effects of CTLA4-Ig treatment on circulating fibrocytes and skin fibroblasts from the same systemic sclerosis patients: an in vitro assay
Source: Arthritis Res Ther. 2018 Jul 27;20:157. doi: 10.1186/s13075-018-1652-6 (PMC6062881; doi:10.1186/s13075-018-1652-6)

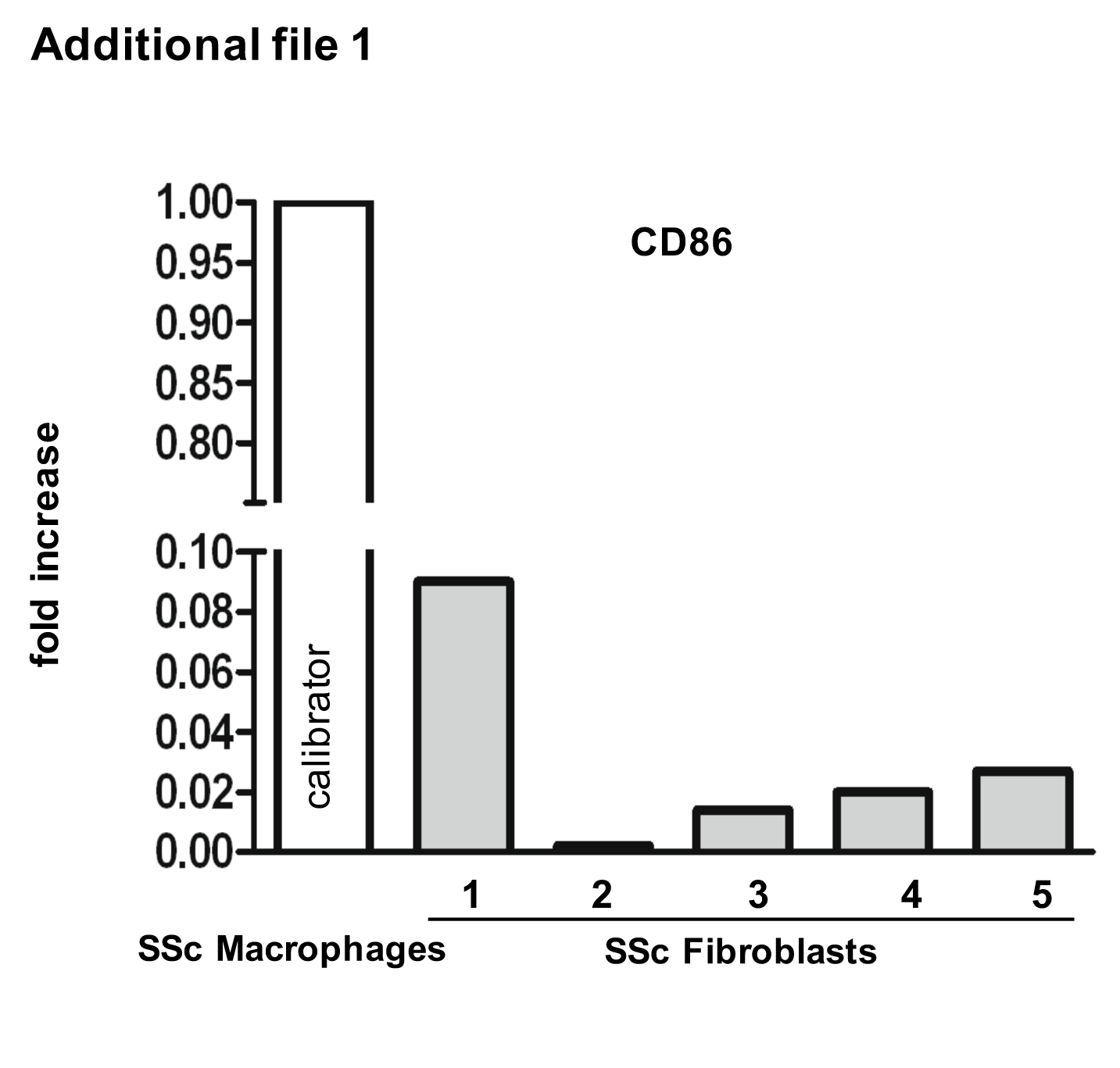

Supplement: Supplementary file 1 — Figure S1. CD86 gene expression levels in cultured SSc fibroblasts. Bar graph of quantitative RT-PCR analysis for CD86 gene expression in cultures of SSc fibroblasts, compared with SSc macrophages, taken as calibrator. (TIF 180 kb) [file 13075_2018_1652_MOESM1_ESM.tif]
